# Subtotal Resection of a Thalamic Glioblastoma Multiforme through Transsylvian Approach

**DOI:** 10.7759/cureus.1662

**Published:** 2017-09-07

**Authors:** Rouzbeh Motiei-Langroudi, Homa Sadeghian, Alireza M. Mohammadi

**Affiliations:** 1 Neurosurgery, Beth Israel Deaconess Medical Center, Harvard Medical School; 2 Radiology, Massachusetts General Hospital/Harvard Medical School; 3 Neurological Institute, Cleveland Clinic

**Keywords:** glioblastoma, thalamus, resection, difficult-to-access

## Abstract

Glioblastoma multiforme (GBM) is a malignant brain tumor with an ominous prognosis. The standard treatment includes maximal safe resection plus adjuvant therapy. Thalamic GBMs, however, are unfavorable for microsurgical removal because of deep location and proximity to critical structures. We present a patient presenting with progressive hemiparesis and decreased consciousness with a large thalamic GBM who underwent subtotal resection through a transsylvian approach. His clinical and neurologic condition improved after surgery and he survived nine months after surgery. This may propose that in selected cases, more aggressive microsurgery for debulking of tumors might have some impact in the final outcome.​​​

## Introduction

Glioblastoma multiforme (GBM) is the most common primary brain tumor in adults [1]. It has an ominous prognosis, as it is a diffuse disease, and even gross total resection (GTR) of visible tumors does not eradicate microscopic disease. The standard treatment includes maximal safe resection if feasible, followed by radiation therapy and chemotherapy, with a median survival of 15 months [2].

Some factors make the prognosis even worse; among them are deep-seated tumors, such as thalamic GBMs. They are considered a great challenge to neurosurgeons, and are unfavorable for microsurgical removal because of the proximity to critical structures, which limits access and reduces extent of resection [3]. For many years, more conservative treatment paradigms have been advocated, including tissue diagnosis by biopsy followed by adjuvant therapy (radiation plus chemotherapy), as the treatment of choice in thalamic GBMs [4].

Some studies, however, have reported subtotal resection (STR), followed by radiation therapy and chemotherapy in the management of these tumors [3, 5-7]. Albeit, the survival benefits of such surgical interventions have not been proven yet [8].

Here, we present a patient diagnosed with a large thalamic glioblastoma who underwent STR through a transsylvian approach. His clinical and neurologic condition improved after surgery.

## Case presentation

**Figure 1:**
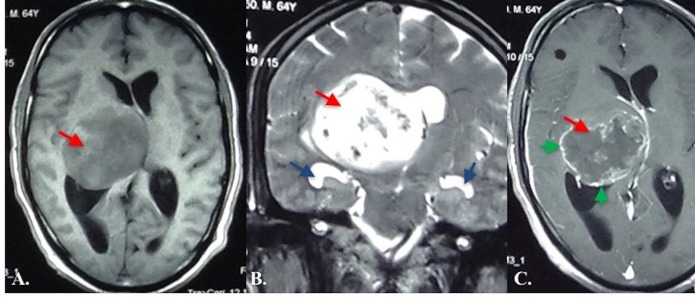
Preoperative Magnetic Resonance Imaging of the Patient T1-weighted (A), T2-weighted (B), and T1 after Gadolinium (C) show a high-grade lesion in right thalamus (red arrow) with peripheral contrast enhancement (green arrow). Note that the patient is getting hydrocephalic as temporal ventricular horns are enlarged (blue arrows).

The patient, a 63-year-old man, presented with decreased consciousness and left hemiparesis (scoring two out of five in motor exam). His family mentioned a history of progressive headache, nausea, vomiting, and decreased motor forces during the past few months. In the computed tomography (CT) scan without contrast, a hypodense mass lesion was observed within the right thalamus. Magnetic resonance imaging (MRI) showed a 5 x 5 x 4.5 cm T1 hypointense, and a T2 hyperintense lesion with strong enhancement, after Gadolinium injection in the right thalamus, with an extension to right ventricle body accompanied by significant edema, hydrocephalus, and midline shift (Figure 1).

**Figure 2:**
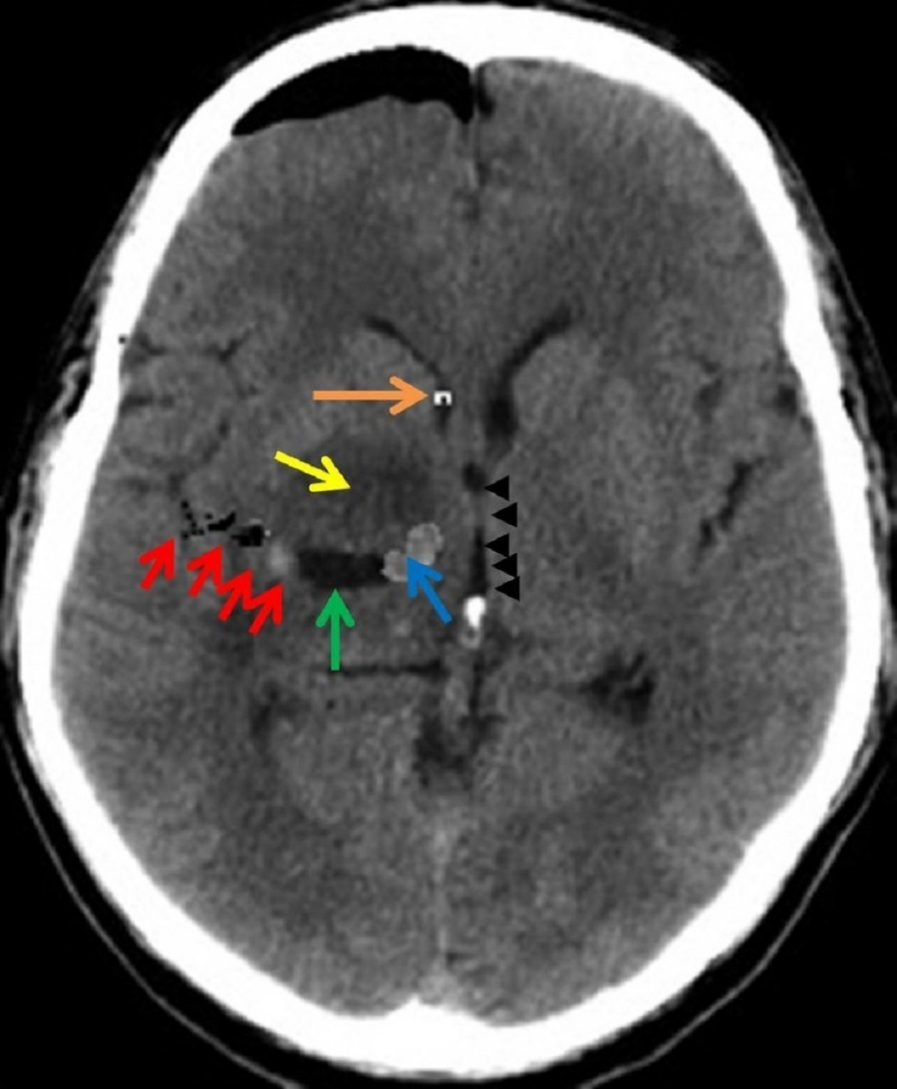
Postoperative Computed Tomography (CT) Scan of Patient Postoperative computed tomography (CT) scan of patient shows the trajectory (red arrows), the resection cavity (green arrow), with some tumor remnant mixed with small hematoma (blue arrow) and edema (yellow arrow). An extraventricular drain (EVD) is visible in lateral ventricle (orange arrow), reducing the hydrocephalus. Note that the midline shift is decreased due to the resection (black arrowheads).

Steroids were started and the patient underwent craniotomy to resect the tumor. In a supine position with the head fixed in a three-point fixation device (Rigid Cranial Fixation Device, Behgaran Teb Co., Tehran, Iran), a right temporoparietal craniotomy was performed. After opening the dura, a standard transsylvian approach was performed under microscopic magnification. Opening the sylvian fissure from lateral to medial, a 2 cm cortisotomy was performed, and the tumor was reached at a 1-2 cm depth. The tumor was poorly defined, soft, and with a yellow to purple color. The tumor was resected in a piecemeal fashion, starting by the core. Resection was more limited in anterolateral and inferior aspects of tumor. After hemostasis, the dura mater, bone, muscle, and skin were closed. An extraventricular diversion (EVD) system was also inserted for the patient from Kocher’s point. By completion of the surgery, the patient was extubated and transferred to the intensive care unit (ICU). After surgery, his decreased consciousness fully recovered, and his hemiparesis slightly improved to three out of five. The postoperative CT showed no surgery-related complications, and the MRI showed a 3 x 2 x 1.5 cm remaining mass (an approximate 90% mass reduction) (Figure 2).

The pathologic examination was consistent with the diagnosis of glioblastoma multiforme (World Health Organization grade four).

The attempt to remove the EVD was unsuccessful due to continued hydrocephalus in brain CT and obtundation; therefore, it was converted to a ventriculo-peritoneal shunt system, CSF Flow-Control valves (Medtronic, Minneapolis, Minnesota, USA) after five days. The patient left the hospital 13 days after admission with complete consciousness, slightly improved hemiparesis (three out of five), and no surgery-related complications. The patient was then referred for adjuvant therapy (radiation therapy plus chemotherapy), which was not performed due to the patient's wish to forego the procedure. 

After nine months, the patient was diagnosed with recurrence based upon symptoms of decreased consciousness and hemiplegia, and imaging. Per family request, the patient underwent second debulking surgery, and eventually died after three weeks due to pulmonary embolism.

All procedures were performed in adherence to regulations of the ethics committee of Bam University of Medical Sciences. The patient (whenever conscious and oriented) and his family gave their written consent for all procedures. 

## Discussion

GBM is classified as a high-grade glioma, and is the most common malignant primary brain tumor [1, 7]. It is extremely invasive, and although attempts to perform GTR have been successful in many cases, it almost never results in microscopic total resection as microscopic tumors always remain, causing a poor prognosis. The standard treatment includes maximal safe resection, especially when the anatomic location of the tumor allows, followed by radiation and chemotherapy with temozolomide. Newer forms of therapy, including immunotherapy and antiangiogenic treatment, have also been proposed. The median survival has not yet exceeded 15 months [2].

Some factors have been proposed to alter the prognosis, including age, presence of preoperative neurologic deficits, and tumor location. Deep-seated tumors in structures like the thalamus, midbrain, or brain stem have always been a great challenge in microsurgery, as GTR is limited in these locations. As a result, less tumor mass can be resected, with the final consequence of a worse prognosis [3]. In supratentorial GBMs in areas other than the thalami or hypothalamus, it has been shown that more extensive tumor removal (GTR vs. partial resection or biopsy only) increases overall and progression-free survival [9].

For many years, more conservative treatment paradigms, including tissue diagnosis by stereotactic or open biopsy, followed by adjuvant therapy (radiation plus chemotherapy) have been advocated as the treatment of choice [4]. Some believe that aggressive surgery to achieve GTR or STR adds no benefit in improving survival, but only increases surgical morbidity and complications [8].

However, the anatomy of the thalamus may allow for the possibility of surgery. The thalamus has a tetrahedron-like shape with three free surfaces; only the ventrolateral border approximates critical anatomical structures, including the internal capsule and subthalamic nucleus, and a surgical approach can be more safely made through the other surfaces [3, 8]. Based on these considerations, there are reports of attempted STR followed by adjuvant therapy in the management of thalamic tumors [3, 5-7]. Steiger, et al. reported 14 patients with thalamic gliomas, 10 with high grade (grade three and four) gliomas upon whom STR or GTR was attempted through a parieto-occipital transventricular approach with an acceptable morbidity profile (two among 14 had new partial hemianopia) [3]. In some other case reports, these attempts have resulted in outstanding results [5, 10]. Kelly suggested surgery (as opposed to stereotactic biopsy followed by adjuvant therapy) in those suffering from an enlarging tumor with significant mass effect, or in those with recurring cysts [6]. Regarding the rarity of studies reporting STR or GTR in thalamic tumors, surgical approaches to this region are not well standardized. Most studies advocate transcallosal or transventricular (whether anterior or posterior) or transcortical (through posterior parietal) lobule approaches as these routes direct the trajectory medial to the internal capsule, arcuate fasciculus, and optic radiation.

In the current study, we reported a patient with a massive thalamus GBM, presenting with progressive symptoms, including decreased consciousness and significant hemiparesis, who underwent STR (>90% resection) through a transsylvian approach. The decision for the operation was made on the basis of the patient’s rapidly progressive neurologic deterioration, which was caused not only due to his hydrocephalus, but also because of his massive lesion and midline shift. We selected transsylvian approach based on certain considerations: 1) on preoperative imaging, the shortest distance between a cortical entry point and tumor surface was achieved through a transsylvian approach, since the distance between depth of sylvian fissure and tumor surface was even shorter than the distance between posterior parietal cortex and lateral ventricle, even in the presence of hydrocephalus; 2) the occurrence of tumor in the right hemisphere, which precluded damage to the language cortices; 3) the tumor could be reached by a posterior trajectory without damage to the internal capsule, though our patient was already hemiparetic before surgery; 4) in patients possessing large infiltrative structures with preoperative major neurologic deficits, many important pathways and structures are already deviated or destructed by the tumor itself.

In our case, it can be assumed that the huge size of tumor had pushed motor fibers anteriorly, if not already destructed by the tumor itself, making a relatively safe corridor through the transsylvian. Given the availability of intraoperative navigation in many settings nowadays, this approach can be deployed with more safety in select cases. Our patient significantly improved after the first surgery and had no surgical complications. However, he failed to pursue adjuvant therapy, and the tumor recurred after nine months, resulting in the demise of the patient. Had he received adjuvant therapy, longer survival would have accomplished. To our knowledge, it is the first report of maximal resection in a thalamic GBM through a transsylvian approach. However, since our study was conducted in the context of a single case, more patients need to be treated and their long-term follow-ups reported, in order to make reliable conclusions.

## Conclusions

GBM is a malignant brain tumor with a poor prognosis. The prognosis gets even worse for certain anatomical locations, including brainstem or deep structures like thalami, as maximal surgical resection is further limited. We treated a large thalamic GBM in a very poor neurologic state and obtained a nine-month survival, even without adjuvant radiation and chemotherapy. The result of this limited experience suggests that in selected cases, more aggressive microsurgery with the aim of STR or GTR can be achieved in the resection of thalamic GBMs with less than expected complications. To achieve this, among many other surgical approaches, transsylvian approach may be considered as a feasible option, in exceptional cases of large tumors with pre-existing major neurologic deficits.

## References

[1] Woehrer A, Bauchet L, Barnholtz-Sloan JS (2014). Glioblastoma survival: has it improved? Evidence from population-based studies. Curr Opin Neurol.

[2] Alifieris C, Trafalis DT (2015). Glioblastoma multiforme: pathogenesis and treatment. Pharmacol Ther.

[3] Steiger HJ, Götz C, Schmid-Elsaesser R (2000). Thalamic astrocytomas: surgical anatomy and results of a pilot series using maximum microsurgical removal. Acta Neurochir (Wien).

[4] Prados MD, Wara WM, Edwards MS (1995). The treatment of brain stem and thalamic gliomas with 78 Gy of hyperfractionated radiation therapy. Int J Radiat Oncol Biol Phys.

[5] Aguilera DG, Tomita T, Rajaram V (2009). Glioblastoma multiforme in a patient with chronic granulomatous disease treated with subtotal resection, radiation, and thalidomide: case report of a long-term survivor. J Pediatr Hematol Oncol.

[6] Kelly PJ (1989). Stereotactic biopsy and resection of thalamic astrocytomas. Neurosurgery.

[7] Nishio S, Morioka T, Suzuki S (1997). Thalamic gliomas: a clinicopathologic analysis of 20 cases with reference to patient age. Acta Neurochir (Wien).

[8] Krouwer HG, Prados MD (1995). Infiltrative astrocytomas of the thalamus. J Neurosurg.

[9] Ushio Y, Kochi M, Hamada J (Tokyo). Effect of surgical removal on survival and quality of life in patients with supratentorial glioblastoma. Neurol Med Chir.

[10] Pollak L, Gur R, Walach N (1997). Clinical determinants of long-term survival in patients with glioblastoma multiforme. Tumori.

